# Comparison of Opioid-Based and Multimodal Analgesic Regimens for Postoperative Pain Management in Gynecological Surgery

**DOI:** 10.7759/cureus.92682

**Published:** 2025-09-18

**Authors:** Ammarah Ahmed, Afnan Amjad

**Affiliations:** 1 Department of Obstetrics and Gynecology, Muhammad Teaching Hospital (MTH) Peshawar, Peshawar, PAK; 2 Department of Anesthesia, Lady Reading Hospital, Medical Teaching Institution (MTI), Peshawar, PAK

**Keywords:** enhanced recovery, multimodal analgesia, patient satisfaction, postoperative pain management, visual analogue scale (vas)

## Abstract

Introduction: Effective postoperative pain management is crucial for patient recovery. Traditional opioids cause significant side effects, including dependency risk. Multimodal analgesia targets multiple pain pathways, reducing opioid consumption while improving outcomes.

Methods: This is a prospective study that was conducted over six months at Muhammad Teaching Hospital, Peshawar, with 100 participants undergoing elective gynecological surgery. Participants were randomly assigned to receive either multimodal analgesia or traditional analgesia. Data were collected on VAS (Visual Analogue Scale) pain scores, opioid consumption, adverse effects, and patient satisfaction levels.

Results: Patients in the multimodal group demonstrated significantly lower VAS pain scores at all time points (one hour: 4.1 ± 0.77 vs. 6.4 ± 0.63, p < 0.01; 24 hours: 2.76 ± 0.55 vs. 4.66 ± 0.47, p < 0.01), reduced opioid consumption (6.4 mg vs. 13 mg in the first 24 hours, p < 0.01), and fewer adverse effects including nausea (10% vs. 26%, p < 0.01) and vomiting (8% vs. 26%, p < 0.01) compared to the traditional group.

Conclusion: Multimodal analgesia is an effective method of pain management that should be offered to postoperative gynecological patients.

## Introduction

Proper pain management after any surgery has an important role in the process of recovery of the patient and satisfaction of the patient [1]. If this pain is not managed properly, it can lead to prolonged hospital stays, which can lead to further complications like wound infection [2]. It also results in decreased satisfaction of the patient [3]. Historically, opioids have been the mainstay of postoperative pain control [4]. However, opioids put the patient at risk of dependency development along with other side effects, including severe nausea and vomiting [5]. To avoid the side effects of opioids, the multimodal analgesia technique was introduced [6]. In this technique, pain relief is managed by aiming at multiple pathways in the pain transmission process, thereby producing a synergistic effect. This is achieved by using a combination of different analgesic medications and techniques [7]. The opioid consumption can be decreased by using the multimodal techniques [8]. Along with effective pain relief, multimodal analgesia hastens functional recovery and decreases the length of hospital stay, thereby lowering the cost of healthcare [9]. 

This study aimed to compare multimodal analgesia versus traditional opioid-based analgesia in patients undergoing elective gynecological surgeries. The primary outcome was postoperative pain control assessed by Visual Analogue Scale (VAS) scores. Secondary outcomes included opioid consumption and adverse effects. We hypothesized that multimodal analgesia would provide superior pain relief with reduced opioid use and fewer side effects.

## Materials and methods

This prospective study was conducted over six months at Muhammad Teaching Hospital in Peshawar, Pakistan, after obtaining approval from the Research Ethics Review Committee (approval no. IRB/13/25). A total of 100 female patients undergoing elective gynecological surgeries via either the vaginal or abdominal route were enrolled after providing informed written consent. Inclusion criteria included patients aged 20 to 60 years, undergoing elective open abdominal or vaginal surgery, with an American Society of Anesthesiologists (ASA) physical status I or II. Participants were randomly assigned to two groups using computer-generated random numbers: one group received opioid analogues for postoperative pain management, while the other was treated with multimodal analgesia.

The multimodal analgesia group received a combination of acetaminophen, regional anesthesia (including nerve blocks such as the transversus abdominis plane (TAP) block), and local anesthetics, whereas the traditional analgesia group received opioid-based analgesia, such as morphine. The primary outcome measured was postoperative pain control. Prior to surgery, the participants were instructed on using the VAS, a 10 cm line ranging from 0 (no pain) to 10 (worst pain imaginable), with scores of 1-4 indicating mild pain, 5-8 moderate pain, and above 8 severe pain. Pain scores were recorded at one, six, 12, and 24 hours postoperatively, along with data on adverse effects and opioid consumption. Data were analyzed using IBM SPSS Statistics for Windows, version 26.0 (IBM Corp., Armonk, NY). Normality and equal variance assumptions were verified before applying independent t-tests for continuous variables and Chi-square tests for categorical variables. A p-value of <0.05 was considered statistically significant.

## Results

The demographic features did not differ significantly between the two groups. The mean age in the traditional group was 51± 6.1, while in the multimodal group, it was 51.46± 4.8. The BMI in the traditional group was 25.6 ± 1.9, while the mean BMI in the multimodal group was 26.80 ± 1.12, as shown in Table 1. 

**Table 1:** Patient demographics

Characteristics	Traditional group (n = 50)	Multimodal group (n = 50)
Mean age (years)	51.00 ± 6.1	51.46 ± 4.8
BMI (kg/m^2^)	25.60 ± 1.9	26.80 ± 1.12
Vaginal surgeries	42%	37%
Abdominal surgeries	58%	63%

In the multimodal group, significantly lower VAS scores were observed at every point as compared to the opioid group, as shown in Table 2. 

**Table 2:** VAS scores in different groups at one, six, 12, and 24 hours postoperatively VAS: Visual Analogue Scale, SD: standard deviation

Time post-surgery	Traditional group (mean ± SD)	Multimodal group (mean ± SD)	p-value
1 hour	6.4 ± 0.63	4.1 ± 0.77	<0.01
6 hours	5.5 ± 0.70	3.3 ± 0.71	<0.01
12 hours	5.24 ± 1.00	3.34 ± 0.71	<0.01
24 hours	4.66 ± 0.47	2.76 ± 0.55	<0.01

An opioid-sparing effect was observed in the multimodal group, which had significantly lower opioid consumption compared to the traditional group. In the first 24 hours postoperatively, the multimodal group consumed 6.4 mg of morphine versus 13 mg in the traditional group (p < 0.01).

The incidence of side effects was markedly reduced in the multimodal group, reflecting improved tolerability and reduced adverse effects, as shown in Table 3.

**Table 3:** Comparison of adverse effects between treatment groups

Adverse effect	Traditional group	Multimodal group	p-value
Nausea	26%	10%	<0.01
Vomiting	26%	8%	<0.01
Diarrhea	22%	6%	<0.01

Our study showed that patients who received the multimodal analgesia had significantly lower VAS scores than those who received only opioid analgesics. VAS pain scores were reduced along with opioid consumption in the multimodal analgesia group. The decreased opioid intake resulted in a lower incidence of common opioid related side effects, including nausea and vomiting.

## Discussion

If the postoperative pain is not managed properly, it may produce a variety of adverse physiological effects in the acute phase and delayed recovery and chronic pain in the long term, thus making optimal pain management mandatory [10].

Opioids have been used as the prime analgesics for post-op pain for ages. However, they come with the cost of adverse effects [11]. To overcome this problem, the multimodal analgesic technique was introduced about a decade ago. The aim was to reduce the opioid-related side effects and improve the efficacy of analgesia [12]. 

In our study, patients in the multimodal group expressed significantly lower VAS scores at every point as compared to the opioid group. Similar results were observed in a Korean study [13] that was conducted by Nam SW et al.

In the case of multimodal analgesia, different analgesic agents are used via different routes to interact with different pain receptors, which leads to achieving an optimal pain relief at a lower dose of analgesics, thus reducing the adverse effects of each of the individual agents [14]. Our study showed a significant decrease in these side effects in the multimodal group as compared to the opioid group. These results are comparable to the findings of a study Ramaseshan AS et al. conducted in 2020 [15]. The significant decrease in the pain relief and reduced opioid consumption demonstrates the competence of multimodal analgesia to offer better pain control while mitigating risks associated with opioid use, namely, nausea and vomiting [16]. Our study demonstrated a significantly reduced opioid consumption in the multimodal group as compared to the opioid group. These results were similar to a study conducted in 2021 by Ackenbom MF et al. [17]. The reduced opioid consumption demonstrates the competence of multimodal analgesia to offer better pain control while mitigating risks associated with opioid use [18].

These factors markedly boost patient satisfaction, contributing to improving clinical outcomes and reducing healthcare expenditure [19].

## Conclusions

The findings of this study highlight the effectiveness of multimodal analgesia in managing postoperative pain in patients undergoing elective gynecological surgeries. Patients who received multimodal analgesia experienced significantly lower pain scores at all measured intervals and required less opioid medication compared to those managed with traditional opioid-based regimens. This reduction in opioid use was also associated with a marked decrease in common opioid-related side effects, such as nausea, vomiting, and diarrhea.

By utilizing a combination of analgesic agents and techniques that target different mechanisms of pain, multimodal analgesia offers a more balanced and tolerable method of pain control. The results of this study support the incorporation of multimodal analgesia into standard postoperative pain management protocols for gynecological surgeries. Broader implementation of this approach could lead to improved clinical outcomes and a safer recovery experience for patients. Further research is encouraged to explore its long-term benefits and application across other surgical disciplines.
